# Bempedoic Acid in Statin-Intolerant Patients: Real-World Implementation Strategies, Cardiovascular Outcomes Beyond the CLEAR Trial, and Combination Therapy Approaches

**DOI:** 10.1007/s11883-026-01483-3

**Published:** 2026-11-05

**Authors:** Carlos Vergara Sanchez, Rodolfo A. Lopes, Michael D. Shapiro

**Affiliations:** 1 Department of Cardiovascular Medicine, Mayo Clinic, Jacksonville, FL USA; 2 Department of Internal Medicine, Memorial Healthcare System, Hollywood, FL USA; 3 Center for Prevention of Cardiovascular Disease, Section on Cardiovascular Medicine, Wake Forest University School of Medicine, Winston-Salem, NC 27157 USA

**Keywords:** Bempedoic acid, Primary prevention, Secondary prevention, ASCVD, Statin intolerance, Lipid management

## Abstract

**Purpose of the Review:**

Statin intolerance remains a significant barrier to achieving lipid goals and reducing atherosclerotic cardiovascular disease (ASCVD) risk. Bempedoic acid (BA), an oral ATP-citrate lyase inhibitor activated predominantly in the liver, offers a non-statin lipid-lowering option with a low propensity for muscle-related adverse effects. This review seeks to discuss BA use in real-world practice.

**Recent Findings:**

This review summarizes BA pharmacology, cardiovascular outcomes, safety, combination strategies, and real-world implementation in individuals who are either statin intolerant or unwilling to take statins. The CLEAR clinical trial series established BA's lipid-lowering effect and safety profile, culminating in CLEAR Outcomes, which further demonstrated fewer major adverse cardiovascular events among patients with established ASCVD or at high cardiovascular risk. Although these findings are compelling, they are limited to this population, and the impact in low- to borderline-risk populations remains unclear.

**Summary:**

In practice, clinicians should assess the potential contribution of the nocebo effect, distinguish partial versus complete statin intolerance, preserve any tolerated statin exposure, define the residual lipid-lowering gap, and align treatment intensity with cardiovascular risk and patient preferences. BA may then be considered within a guideline-directed framework that accounts for treatment access, tolerability, and shared decision-making. Combination therapy with BA and ezetimibe represents a practical oral option, whereas PCSK9-targeted therapy may be preferred when the gap to lipid goals is sufficiently large or the patient is at high ASCVD risk. Clinicians should also provide risk-based counseling and monitoring for potential adverse effects, including hyperuricemia, gout, and tendon injury. Overall, BA represents a valuable addition to the lipid-lowering armamentarium.

**Graphical Abstract:**

**Figure:**
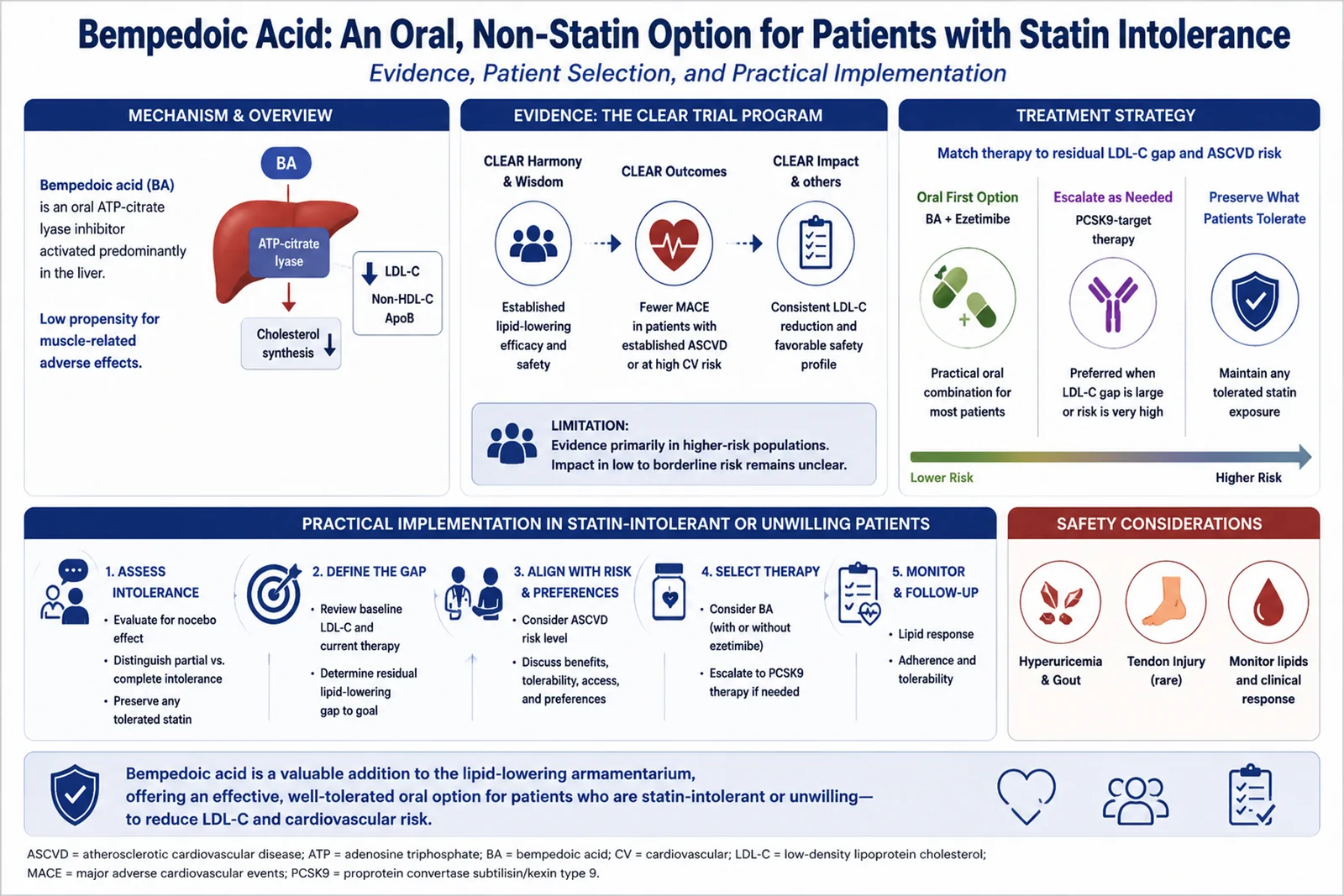

## Introduction

Cardiovascular disease (CVD) remains the leading cause of morbidity and mortality worldwide and represents a substantial global burden. Despite reductions in age-standardized mortality, the global burden of CVD has nearly doubled, driven in part by modifiable risk factors such as elevated low-density lipoprotein cholesterol (LDL-C) levels [1].

Apolipoprotein B (apoB)-containing lipoproteins, particularly LDL-C, have a dose-dependent role in the development and progression of atherosclerotic cardiovascular disease (ASCVD). The concept of "cholesterol-years" as a determinant of lifetime ASCVD risk reinforces the principle that lower LDL-C levels achieved earlier and maintained longer confer greater benefit, leading guidelines to recommend aggressive, risk-based LDL-C lowering targets across primary and secondary prevention populations [2–5]. Statins remain the foundation of lipid-lowering therapy because of their established efficacy in reducing circulating apoB-containing particles, stabilizing plaque, and improving cardiovascular outcomes at low cost [6, 7].

However, statin intolerance remains a clinically relevant concern, contributing to treatment discontinuation and suboptimal LDL-C control, thereby perpetuating residual cardiovascular risk [8]. While a proportion of reported symptoms may be secondary to a nocebo effect, the impact on adherence in real-world practice is substantial [9]. Alternative non-statin lipid-lowering therapies have further expanded treatment options, but each has important limitations. Ezetimibe provides modest LDL-C reduction, whereas proprotein convertase subtilisin/kexin type 9 inhibitors (PCSK9i) are limited by higher cost, restricted accessibility, and route of administration [10–12].

Bempedoic acid (BA) is an oral non-statin therapy whose activation occurs in the liver, thereby limiting exposure of skeletal muscle to the active drug and reducing the potential for muscle-related adverse effects [13]. The CLEAR Outcomes trial demonstrated that BA lowers LDL-C levels and reduces major adverse cardiovascular events (MACE) in statin-intolerant and/or unwilling patients with established ASCVD or at high cardiovascular risk, supporting its role as an effective treatment option [14].

Questions remain regarding the optimal use of BA within the contemporary lipid-management landscape and its position relative to other non-statin therapies. This review examines its role in statin-intolerant patients and its integration into risk-based, combination lipid-lowering strategies, with emphasis on cardiovascular outcomes and real-world application.

## Statin Intolerance in Clinical Practice

Statin intolerance has a broad clinical spectrum and is an important challenge because statins are the most extensively validated lipid-lowering therapy. Whether symptoms are pharmacologically mediated, influenced by a nocebo effect, or multifactorial, intolerance is one of the leading causes of discontinuation and undertreatment of patients at high ASCVD risk [15].

The National Lipid Association (NLA) defines statin intolerance as “*one or more adverse effects associated with statin therapy which resolve with dose reduction or discontinuation and may be classified as complete intolerance to any statin dose or partial intolerance*.” [15] At least two statins should be attempted, including one at the lowest approved daily dose. Partial intolerance permits some tolerated statin exposure, whereas complete intolerance precludes any acceptable regimen.

The definition extends beyond statin-associated muscle symptoms (SAMS), which often represent the most common manifestation. Statin intolerance encompasses a range of multiorgan symptoms, whether pharmacologically mediated or not, that lead patients to self-identify as intolerant (Table 1) [16]. Its prevalence varies from 3 to 30%, influenced by population characteristics, while meta-analysis-derived data suggest an overall prevalence of 9.1% [17]. Although the SAMSON study suggested that many reported statin-associated adverse effects may be attributable to the nocebo effect [18], statin intolerance remains a significant barrier to achieving guideline-directed lipid targets. Consequently, effective alternative lipid-lowering therapies have become increasingly important, among which BA has emerged as a valuable treatment option.

**Table 1:** Multiorgan-System Manifestations Associated with Statin Therapy^15^. Skeletal muscle manifestations are the most commonly reported adverse effects associated with statin therapy and range from mild myalgias to rare but severe complications, such as rhabdomyolysis and anti-HMGCR immune-mediated necrotizing myopathy. Hepatic and metabolic effects are less common and are generally mild or dose-dependent. Clinically significant hepatotoxicity is rare. Reported neurocognitive effects have yielded inconsistent findings and may, in part, reflect nocebo effects or competing etiologies

Skeletal Muscle	Hepatic	Metabolic
Myalgia	Aminotransferase elevation	Dysglycemia
Myopathy	Severe hepatotoxicity (rare)	New-onset diabetes
CK elevation		
Anti-HMGCR immune-mediated necrotizing myopathy		
Rhabdomyolysis		

Most statin-associated muscle symptoms occur without creatine kinase elevation and are reversible with dose reduction or discontinuation

The CLEAR Outcomes trial used a broader definition of statin intolerance. Participants were eligible if they were either unable or unwilling to take statin therapy, thereby including individuals with self-reported statin intolerance [14]. Although broader than the NLA definition, this may better capture real-world clinical practice, where many patients discontinue statins without fulfilling formal definitions of statin intolerance.

## Bempedoic acid: Mechanism, Pharmacology, and Practical Use

**Fig. 1:**
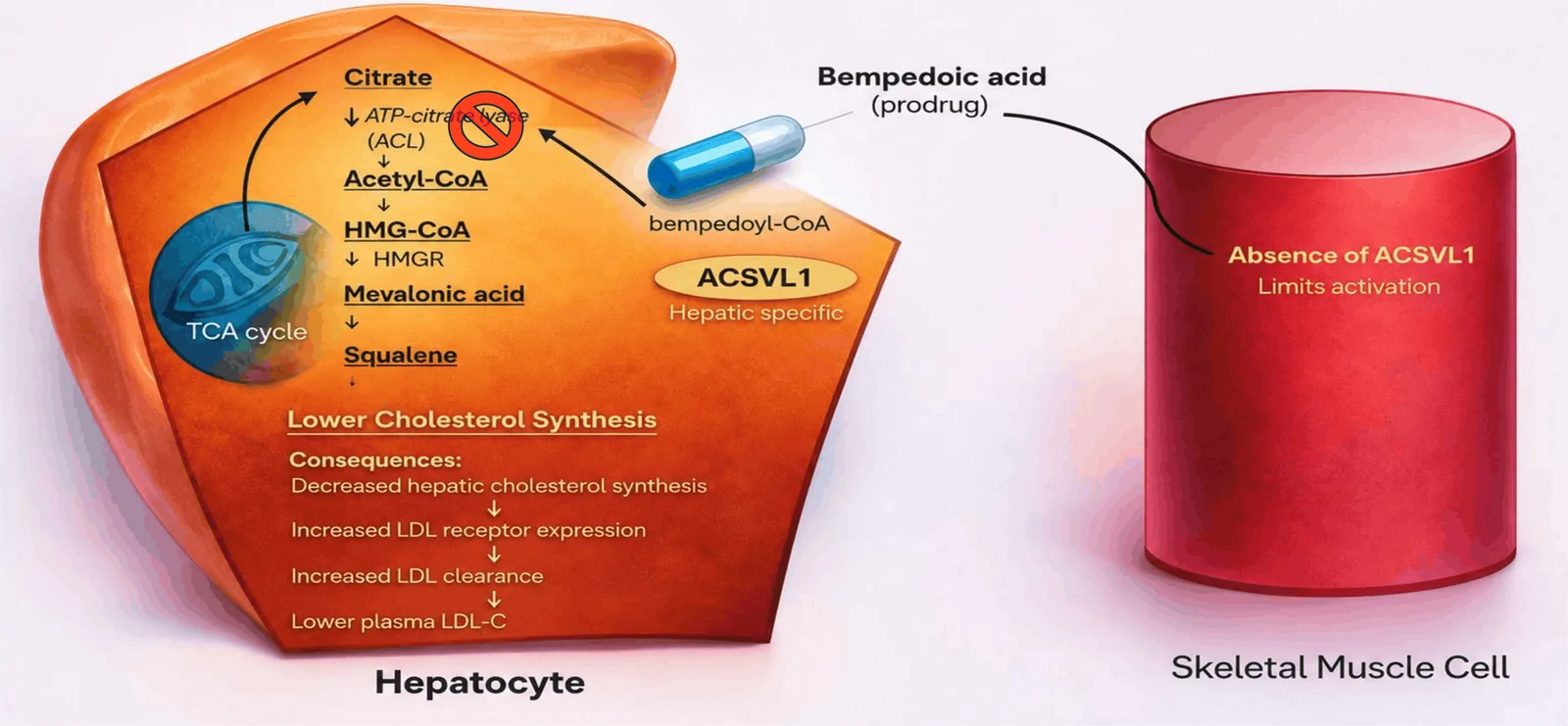
Mechanism of action bempedoic acid within the hepatocyte

BA is a prodrug activated within hepatocytes by very long-chain acyl-CoA synthetase 1 (ACSVL1), an enzyme present exclusively in these cells. This liver-specific activation likely contributes to the lower incidence of muscle-related adverse effects observed with BA compared with statins [19]. Once in its active form, BA inhibits ATP-citrate lyase, the enzyme responsible for catalyzing the conversion of citrate to acetyl-CoA in the liver. This inhibition reduces intrahepatic cholesterol synthesis, acting earlier in the cholesterol synthesis pathway than statins, which target HMG-CoA reductase (Fig. 1).

**Fig. 2:**
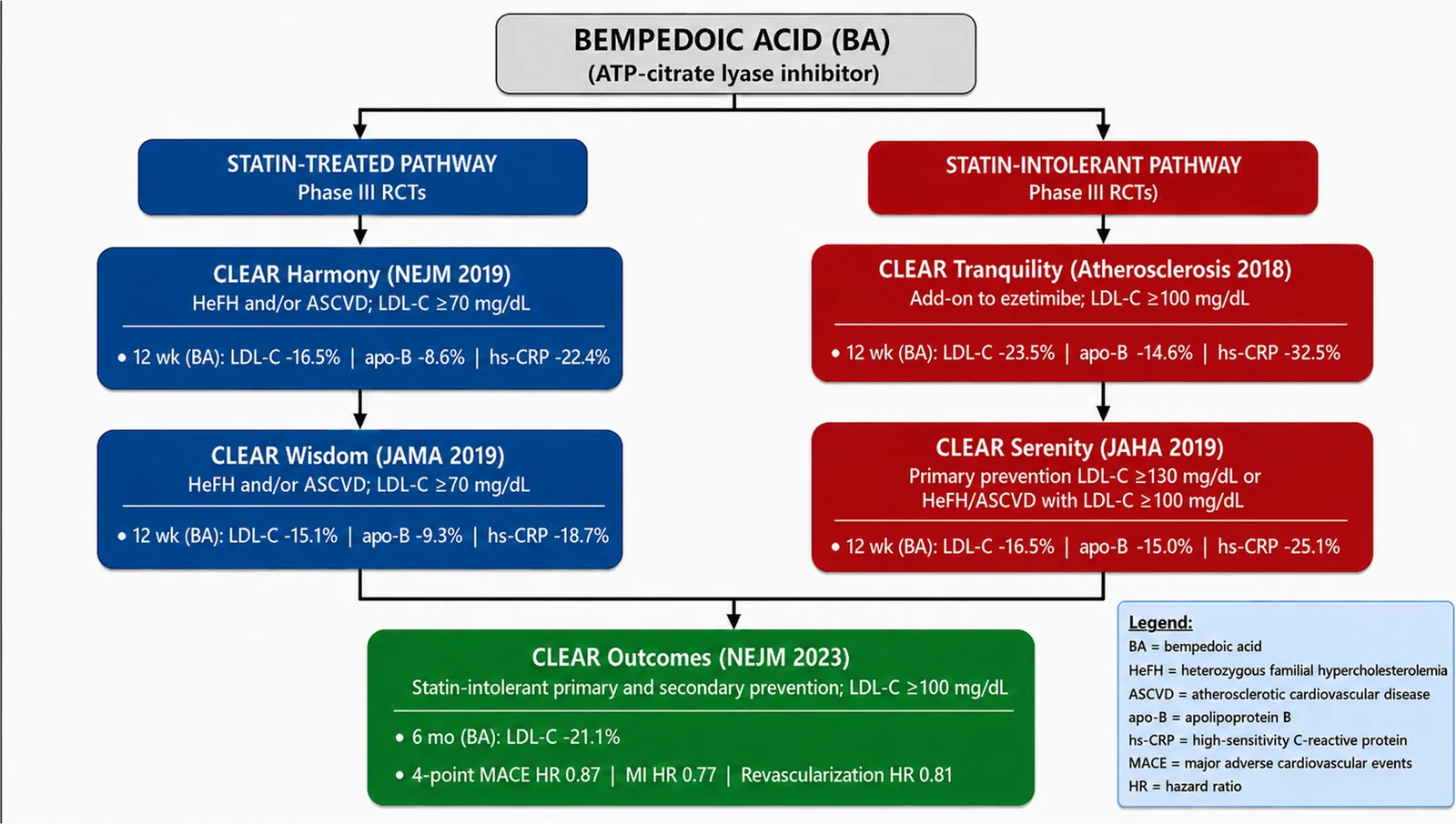
CLEAR trial program. Four phase III randomized clinical trials, leading up to the landmark CLEAR outcomes trial

BA is administered orally at 180 mg once daily, either alone or as a fixed-dose combination with ezetimibe 10 mg. The CLEAR clinical trial program evaluated lipid efficacy, safety, and cardiovascular outcomes across statin-treated and statin-intolerant populations (Fig. 2) [4, 5, 14, 20–24].

## A CLEAR Path Towards Benefit

The CLEAR phase III program included four pivotal lipid-lowering trials. CLEAR Harmony and CLEAR Wisdom enrolled patients with ASCVD and/or heterozygous familial hypercholesterolemia receiving stable maximally tolerated statin therapy, although CLEAR Wisdom had roughly 10% of participants on only non-statin lipid-lowering therapy or no therapy at all. CLEAR Tranquility and CLEAR Serenity enrolled patients deemed to be statin intolerant; CLEAR Tranquility enrolled those also receiving no more than low-dose statin therapy. Across these studies, BA reduced LDL-C, apoB, and high-sensitivity C-reactive protein (hs-CRP) relative to placebo, with generally comparable overall treatment-emergent adverse-event rates (Fig. 2) [14, 21–24]. The initial program provided valuable information regarding the effects of BA on LDL-C reduction in patients receiving maximally tolerated statin therapy as well as in those not taking statins. In pooled analyses, BA reduced LDL-C levels by 18% and 24% in these groups, respectively [25]. These findings supported BA as an important adjunctive oral lipid-lowering therapy when used in combination with statins and as an oral alternative for patients with statin intolerance, providing modest but clinically meaningful LDL-C reductions.

This program culminated in CLEAR Outcomes, a cardiovascular outcomes trial that randomized 13,970 participants aged 18–85 years with LDL-C ≥ 100 mg/dL and either established ASCVD or a high risk of a cardiovascular event. Eligible patients were unable or unwilling to take guideline-recommended doses of statins because of an adverse effect that began or worsened during statin treatment and resolved or improved after statin discontinuation [14]. Participants received BA 180 mg once daily or placebo and were followed for a median of 40.6 months. The four-component primary endpoint of major adverse cardiovascular events (MACE − 4) comprised cardiovascular death, nonfatal myocardial infarction (MI), stroke, and coronary revascularization.

The primary endpoint (MACE − 4) occurred in 11.7% (819 of 6,992 participants) of patients receiving BA and 13.3% (927 of 6,978 participants) of those receiving placebo (hazard ratio [HR], 0.87; 95% CI, 0.79–0.96; P = 0.004), corresponding to an absolute risk reduction (ARR) of 1.6% and a number needed to treat (NNT) of 63. At 6 months, the placebo-corrected difference in LDL-C reduction was 21.1%, with an absolute between-group difference of 29.2 mg/dL (0.76 mmol/L), in favor of BA. The between-group difference in the median percentage change in hs-CRP was − 21.6%. BA also reduced three-component MACE (MACE − 3), comprising cardiovascular death, nonfatal MI, and stroke (8.2% vs 9.5%; HR, 0.85; 95% CI, 0.76–0.96; P = 0.006), fatal or nonfatal MI (3.7% vs 4.8%; HR, 0.77; 95% CI, 0.66–0.91; P = 0.002), and coronary revascularization (6.2% vs 7.6%; HR, 0.81; 95% CI, 0.72–0.92; P = 0.001). However, BA did not significantly reduce stroke, cardiovascular mortality, or all-cause mortality (Table 2) [14, 26]. Overall adverse-event, serious adverse-event, and treatment-discontinuation rates did not differ meaningfully between groups. Despite this, hyperuricemia, gout, hepatic enzyme elevations, cholelithiasis, and renal adverse events occurred more frequently with BA [14].

**Table 2:** CLEAR Outcomes trial design, population, efficacy, and safety results

CLEAR Outcomes Trial^14,22^ (NCT02993406)	
Design	Multicenter, double-blind, randomized, placebo-controlled trial conducted at 1250 sites in 32 countries
Population	Adults aged 18–85 years with LDL-C ≥ 100 mg/dL despite optimized lipid-lowering therapy who were unable or unwilling to take statins due to unacceptable adverse effects*. Participants had either established cardiovascular disease (secondary prevention) or were at high cardiovascular risk (primary prevention).**
Sample size	13,970 randomized: 6992 to bempedoic acid and 6978 to placebo
Intervention	Bempedoic acid 180 mg orally once daily
Comparator	Matching placebo
Median follow-up	40.6 months
Baseline population highlights	Mean age 65.5 years; 48.2% women; 69.9% with prior cardiovascular event; 45.6% had diabetes; mean baseline LDL-C 139.0 mg/dL; 22.7% were taking a low-intensity statin and 11.5% ezetimibe at baseline
Primary endpoint	4-component MACE: cardiovascular death, nonfatal MI, nonfatal stroke, or coronary revascularization
Primary result	Primary endpoint occurred in 11.7% with bempedoic acid vs 13.3% with placebo HR 0.87 (95% CI, 0.79–0.96), P = 0.004
Key secondary results	3-component MACE^#^: 8.2% vs 9.5%, HR 0.85, P = 0.006; fatal/nonfatal MI: 3.7% vs 4.8%, HR 0.77, P = 0.002; coronary revascularization: 6.2% vs 7.6%, HR 0.81, P = 0.001
Neutral outcomes	No significant reduction in fatal/nonfatal stroke, cardiovascular death, or all-cause death
Lipid/biomarker effects	At 6 months, LDL-C reduction was greater by 29.2 mg/dL (0.76 mmol/l), with an observed difference of 21.1% favoring bempedoic acid; hs-CRP median percent change also fell 21.6% with bempedoic acid
Safety signals	Bempedoic acid was associated with higher rates of hyperuricemia (10.9% vs 5.6%), gout (3.1% vs 2.1%), renal impairment (11.5% vs 8.6%), elevation of hepatic enzymes (4.5% vs 3%) and cholelithiasis (2.2% vs 1.2%)
Bottom line	In statin-intolerant patients, bempedoic acid reduced MACE, driven mainly by fewer non-fatal MI and need for coronary revascularization events

^*^Statin intolerance that started or increased during statin therapy and resolved or improved when statin therapy was discontinued, resulting in an inability to tolerate either: a. Two or more statins at any dose, b. One statin at any dose and unwilling to attempt a second statin or advised by a physician not to attempt a second statin.^2^** High-risk primary prevention, defined by (i) Reynolds risk score > 30% or a SCORE risk score > 7.5% over 10 years, (ii) coronary artery calcium score > 400 Agatston units or (iii) type 1 or type 2 diabetes aged > 65 years in women and > 60 years in men (2). **#** Cardiovascular death, nonfatal myocardial infarction, or nonfatal stroke

MACE denotes major adverse cardiovascular events; MI, myocardial infarction; LDL-C, low-density lipoprotein cholesterol; hs-CRP, high-sensitivity C-reactive protein

## Beyond the Primary Results

Several prespecified subgroup analyses were subsequently published and are worth highlighting. Hispanic/Latinx participants numbered 2,333, or approximately 17% of the CLEAR Outcomes cohort. Most were from Latin America (73%), followed by North America (23%). Efficacy and tolerability were comparable with those in non-Hispanic participants. At 6-months, placebo-corrected changes from baseline in LDL-C − 21.0% (− 27.2 mg/dL) among Hispanic/Latinx participants and − 21.2% (− 29.7 mg/dL) among non-Hispanic/Latinx participants. Furthermore, there was no evidence that the relative treatment effect differed by patient ethnicity (interaction P = 0.35) [27]. CLEAR Outcomes also included the largest female representation (6,740 women [48.2% of the cohort]) to date among lipid-lowering trials. A pooled analysis of the four phase III trials identified a greater placebo-corrected LDL-C reduction in women than in men, particularly in the ASCVD and/or heterozygous familial hypercholesterolemia “on-statins” pool (− 21.2% vs − 17.4%; interaction P = 0.044). The corresponding difference was not statistically significant in the low-dose or no-statin pool (− 27.7% vs − 22.1%; interaction P = 0.079) [28, 29]. Female sex was also independently associated with achieving a ≥ 30% LDL-C reduction with BA [29]. However, in the sex-stratified analysis of CLEAR Outcomes, although female participants showed lower cumulative incidences of MACE − 4 and MACE − 3 than male participants, the treatment-by-sex interactions were not significant ((P = 0.82 and P = 0.84, respectively). Analyzing women randomized to BA or placebo, the HR was 0.89 (95% CI, 0.75–1.04), potentially because of lower event rates within this group. Nevertheless, overall findings support similar efficacy and safety across sexes [30].

The prespecified primary-prevention analysis included 4,206 participants (30.1% of the total study population) and demonstrated encouraging results. The mean age was 68 years, 59% were women, and 66% had diabetes. Participants were statin intolerant or unwilling to take statins and deemed at high-risk if they had LDL-C ≥ 100 mg/dL, and at least one high-risk criteria: a Reynolds Risk Score > 30% or 10-year SCORE risk > 7.5%, a coronary artery calcium (CAC) score > 400 Agatston units, or type 1 or type 2 diabetes in women older than 65 years or men older than 60 years. Over a median follow-up of 39.9 months, MACE − 4 occurred in 5.3% of participants receiving BA versus 7.6% receiving placebo, corresponding to an adjusted HR of 0.70 (95% CI, 0.55–0.89; P = 0.002), an absolute risk reduction (ARR) of 2.3%, and a number needed to treat (NNT) of 43. Point estimates favored BA for MI (HR, 0.61; 95% CI, 0.39–0.98), cardiovascular death (HR, 0.61; 95% CI, 0.41–0.92), and all-cause mortality (HR, 0.73; 95% CI, 0.54–0.98). However, the authors noted that these analyses were not adjusted for multiplicity and were considered hypothesis-generating [31]. Most importantly, these findings apply specifically to the selected high-risk population; CLEAR Outcomes did not evaluate BA in a general borderline- or intermediate-risk primary-prevention population.

A prespecified subgroup analysis based on body mass index (BMI) included 6,179 participants (44.2%) with obesity (BMI ≥ 30 kg/m^2^). In this subgroup, 68% had ASCVD and 56% had diabetes. MACE − 4 occurred in 11.6% of participants receiving BA and 14.8% receiving placebo (HR, 0.77; 95% CI, 0.67–0.89), corresponding to a 3.2% ARR and NNT of 31. At 6 months, the placebo-corrected reductions in LDL-C and hs-CRP were 22.5% and 23.2%, respectively. Nevertheless, the interaction across BMI categories was not significant, and findings are best considered as hypothesis-generating given the inherent limitations of the analyses and insufficient statistical power to detect treatment effect differences through BMI strata [32].

In addition, BA also demonstrated consistent efficacy across the glycemic spectrum. Another prespecified analysis stratified by baseline glycemic status and included 6,373 participants with diabetes, 5,796 with prediabetes, and 1,801 with normoglycemia. Among participants with diabetes, BA reduced MACE − 4 (HR, 0.83; 95% CI, 0.72–0.95), but there was no statistical evidence that the relative MACE − 4 differed across glycemia status (interaction P = 0.42). Moreover, among participants without diabetes, the incidence of new-onset diabetes and HbA1C were similar between groups. Thus, BA did not increase new-onset diabetes or worsen glycemic measures during the median 3.4-year follow-up [33].

Finally, a post hoc secondary analysis evaluated composite cardiovascular outcomes across baseline eGFR categories of ≥ 90, 60 to < 90, 45 to < 60, and 30 to < 45 mL/min/1.73m^2^; screening eGFR ≥ 30 mL/min/1.73m^2^ was required for enrollment. The effect of BA on MACE − 4 was consistent across the categorical eGFR strata (interaction P = 0.93) and when eGFR was analyzed continuously (interaction P = 0.95). In the lowest eGFR category, which included only 574 participants, the MACE − 4 HR was 0.96 (95% CI, 0.66–1.38), reflecting substantial imprecision. Serious adverse events became more frequent as eGFR declined in both treatment groups but were roughly similar between BA and placebo within each eGFR category; the laboratory changes in creatinine and uric acid associated with BA were also reported to be consistent across kidney-function categories. No renal dose adjustment is required in patients with mild or moderate renal impairment; however, evidence remains limited in those with more severe renal impairment (stages 4–5; eGFR < 30 mL/min/1.73 m^2^) or end-stage kidney disease [34]. Additional clinical monitoring may therefore be warranted in these populations, although a reduced eGFR alone is not currently a contraindication to BA therapy.

## Safety and Monitoring in Clinical Practice

Hyperuricemia is among the principal concerns with BA. Although, the increase in serum uric acid was modest. In the CLEAR Outcomes trial, the mean change from baseline at 6 months in the BA group was + 0.76 ± 1.2 mg/dL. This increase often occurs within the first 4 weeks of treatment; nevertheless, it increases the risk of precipitating gout, particularly in individuals at higher risk. Gout attacks were described in 3.1% of patients receiving BA, compared with 2.1% of those receiving placebo. As previously described, patients at increased risk included those with pre-existing hyperuricemia, a history of gout, renal dysfunction, or other factors associated with increased uric acid levels.

Before initiating treatment, clinicians should assess the patient’s history of gout and related symptoms. A baseline serum uric acid measurement should be considered in patients at increased risk and repeated after treatment initiation or during follow-up as clinically indicated. Patients should be advised to report acute joint pain, erythema, warmth, or swelling. Clinically significant hyperuricemia or gout should prompt evaluation of contributing factors and management according to current gout-treatment guidelines. Recurrent or difficult-to-control gout may warrant discontinuation of BA and selection of an alternative lipid-lowering therapy [35].

Another concern is tendon rupture, an uncommon but clinically important potential adverse effect of BA. Reported cases have most frequently involved the rotator cuff, biceps tendon, or Achilles tendon. In the CLEAR Outcomes trial, adjudicated tendon rupture occurred in 1.2% of the BA group compared to 0.9% of the placebo group. The risk tends to be greater in adults older than 60 years; patients with renal dysfunction or a history of tendon disorders or rupture; and those receiving systemic corticosteroids or fluoroquinolones. Accordingly, BA should be used cautiously when multiple risk factors coexist, and an alternative lipid-lowering agent should be considered, especially in patients with a history of tendon disorders or rupture [35].

Given the well established benefits of physical activity in reducing cardiovascular-related mortality, [36] the potential risk of tendon injury with BA should not preclude implementation of guideline-directed physical activity recommendations. However, clinicians should advise patients receiving BA to report new tendon pain, swelling, or inflammation promptly and to rest the affected limb. BA should be discontinued if tendinitis is suspected and most certainly if a tendon rupture occurs. These precautions should be reassessed whenever systemic corticosteroid or fluoroquinolone therapy is initiated during BA treatment [35].

Furthermore, transient elevations in liver aminotransferases may occur and should be noted. Although mostly asymptomatic, liver aminotransferase elevations were more prevalent in the BA group than in the placebo group in CLEAR Outcomes (4.5% vs. 3.0%). Moreover, BA was associated with a higher incidence of aminotransferase elevations > 3 × the upper limit of normal (ULN) for both alanine aminotransferase (1.2% vs. 0.8%) and aspartate aminotransferase (1.1% vs 0.6%). Similarly, a higher rate of cholelithiasis in the BA group was uniquely identified in this trial, with nearly twice as many events as in the placebo group (152 events [2.2%] vs. 81 events [1.2%]). A recent molecular analysis of the pathophysiological mechanism underlying this finding showed BA affects several pathways that culminate in lithogenicity, including increased expression of genes related to cholesterol efflux, increased canalicular cholesterol concentration; decreased bile acid synthesis and decreased hepatic bile acid uptake [37].

Lastly, BA has been linked to increased serum creatinine levels, raising concerns about potential renal injury. In CLEAR Outcomes, the investigators included a renal-specific adverse-event outcome because of the known potential for BA to cause minor, reversible changes in creatinine and blood urea nitrogen (BUN). In the trial, renal impairment was reported more frequently in the BA group than in the placebo group (11.5% vs. 8.6%). However, the creatinine increase associated with BA is best interpreted as a small, transporter-mediated laboratory effect rather than established intrinsic nephrotoxicity. BA is proposed to weakly inhibit organic anion transport 2 (OAT2), reducing proximal tubular secretion of creatinine. Consequently this effect raises serum creatinine while lowering creatinine-based eGFR estimates, despite unchanged glomerular filtration [38]. Nevertheless, clinicians should be mindful of this expected effect when considering BA use. A post-hoc analysis of CLEAR Outcomes showed overall safety in chronic kidney disease (CKD) among participants with eGFR ≥ 30 min/1.73 ­m^2^, whereas individuals in CKD stages 4–5 or end-stage renal disease were excluded from the CLEAR series trials [34].

## Approach to Therapies for the Statin-Intolerant Patient

Contemporary clinical trials have demonstrated that adding non-statin agents, such as ezetimibe and PCSK9 directed therapies, to background statin produces incremental LDL-C-lowering effects and reduces cardiovascular events across diverse patient populations [39–41].Accordingly, current guidelines use risk-based percentage reductions and absolute thresholds for LDL-C, non-HDL-C, apoB across the spectrum of ASCVD risk and favor combination therapy when a single agent is unlikely to close the therapeutic gap, typically with a statin as the backbone regimen, with or without an additional non-statin lipid lowering agent [4, 5, 42, 43].

**Fig. 3:**
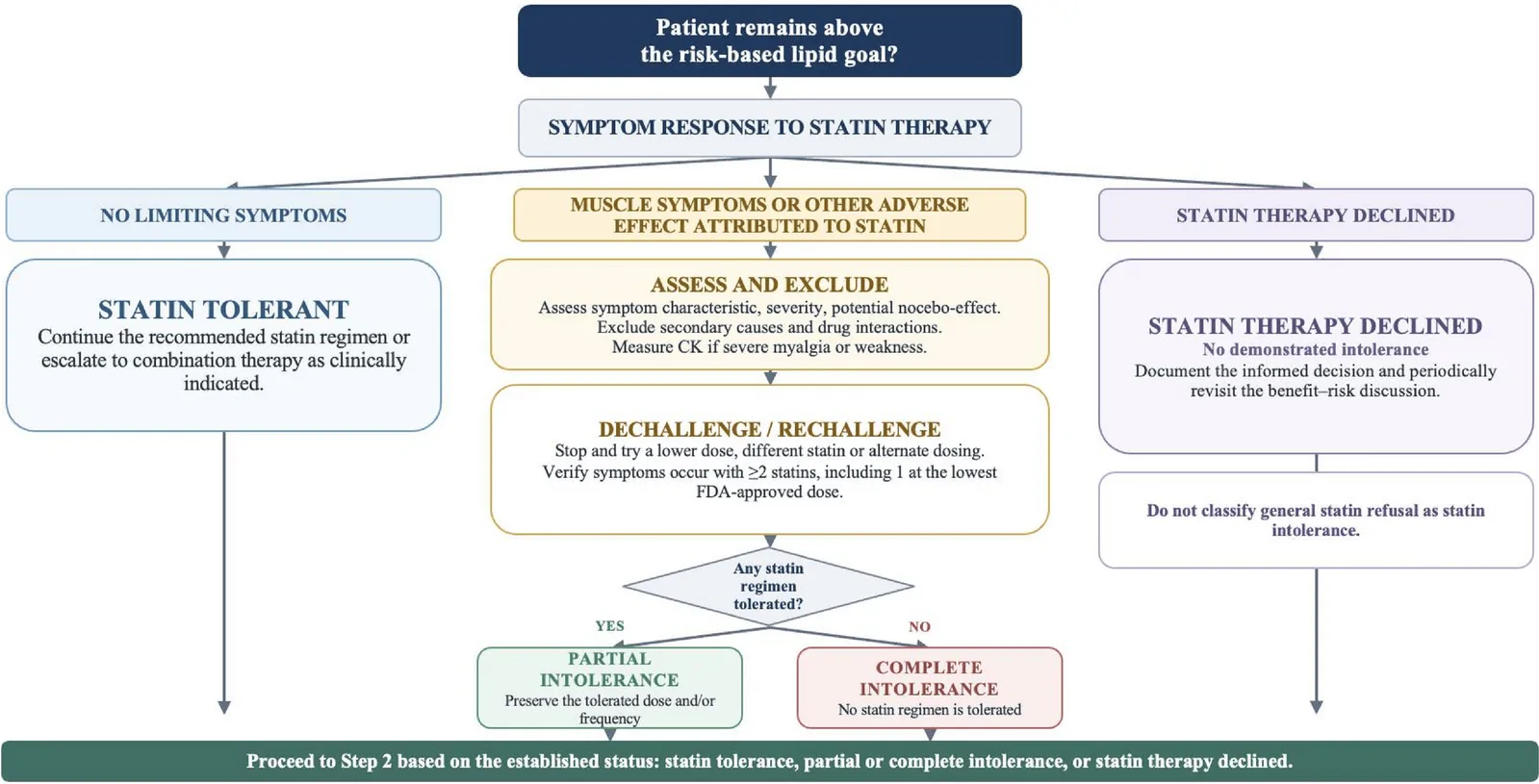
Establishing statin status before selection of non-statin therapy. Reported adverse effects and informed statin refusal are separate pathways; intolerance is classified only after evaluation and rechallenge

The first step in management is to establish the patient's risk-based lipoprotein goal and determine the response intensity required to achieve it. Patients without limiting symptoms should continue the recommended statin regimen or escalate to combination therapy as clinically indicated. A common clinical scenario, however, involves a patient who remains above the recommended treatment goals because adverse effects preclude statin use or intensification. For muscle symptoms or another adverse effect attributed to a statin, clinicians should characterize the symptoms and severity, consider a potential nocebo contribution, exclude secondary causes and reversible contributors, review drug interactions, and measure creatine kinase (CK) when severe myalgia or weakness is present. A rechallenge strategy should test a lower dose, a different statin, or alternate dosing (i.e., intermittent strategy). Intolerance should be established only after symptoms recur during trials of at least two different statins, including one at the lowest FDA-approved dose. If any regimen is tolerated, that dose and/or frequency should be preserved, and the patient should be classified as having partial intolerance, whereas complete intolerance denotes the inability to tolerate any statin regimen. Declining statin therapy without demonstrated intolerance is a distinct scenario in which the clinician should document the informed decision, periodically revisit the benefit–risk discussion, and avoid rapidly classifying general refusal as statin intolerance. (Fig. 3).

**Fig. 4:**
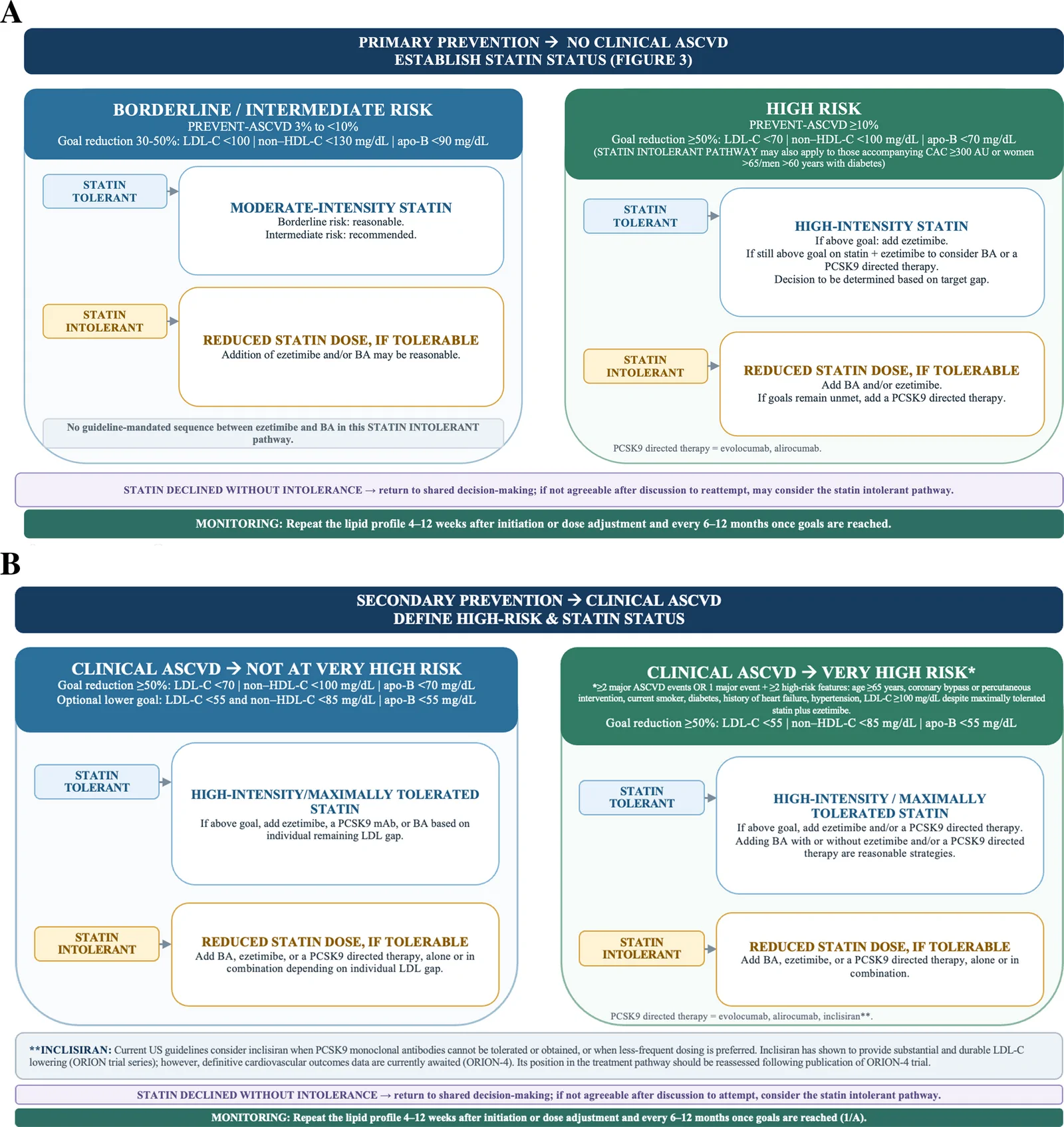
A. Bempedoic Acid in Primary Prevention: 2026 ACC/AHA-Aligned Pathways. Treatment is selected after statin status is established; †CLEAR Outcomes remains as the evidence anchor and not a treatment category. †CLEAR Outcomes supports bempedoic acid (BA) use in selected high-risk primary-prevention patients with adverse-effect–related statin intolerance or trial-defined unwillingness referred specifically to unwillingness to attempt a second statin after experiencing an adverse effect, rather than general statin refusal. Eligible participants were 18–85 years of age, had no prior cardiovascular event, had LDL-C ≥ 100 mg/dL, and met at least one additional high-risk criterion: Reynolds Risk Score > 30%, 10-year SCORE > 7.5%, CAC > 400 AU, or type 1 or type 2 diabetes in women > 65 or men > 60 years. **B**. Bempedoic Acid in Secondary Prevention: 2026 ACC/AHA–Aligned Pathways. Therapy is directed by both very-high-risk ASCVD status and the statin status established in Fig. 3

Once statin status is established, the residual lipid-lowering gap should be quantified within the appropriate primary- or secondary-prevention category, using LDL-C, non–HDL-C and, when available or clinically appropriate, apoB goals. In the U.S. algorithm, borderline/intermediate primary-prevention risk (10-year PREVENT-ASCVD 3% to < 10%) carries a 30%–50% reduction target and goals of LDL-C < 100 mg/dL, non–HDL-C < 130 mg/dL, and apoB < 90 mg/dL; high risk (PREVENT-ASCVD ≥ 10%) carries a ≥ 50% reduction target and goals of < 70 mg/dL, < 100 mg/dL, and < 70 mg/dL, respectively [4]. Statin-intolerant patients can also be classified as high-risk, within primary-prevention context, when identified with a CAC ≥ 300 AU or diabetes in women > 65 years or men > 60 years (Fig. 4A). In secondary prevention, clinical ASCVD deemed not very high risk carries a ≥ 50% reduction target and goals of LDL-C < 70 mg/dL, non–HDL-C < 100 mg/dL, and apoB < 70 mg/dL, with optional lower goals of < 55 mg/dL, < 85 mg/dL, and < 55 mg/dL, respectively. Conversely, in patients with very-high-risk ASCVD, the more intensive target goals of < 55 mg/dL, < 85 mg/dL, and < 55 mg/dL, respectively, are recommended. Very-high-risk status should be assigned using the guideline criteria summarized in Fig. 4B [4, 44].

Treatment selection should account for the magnitude of LDL-C reduction required, ASCVD risk, preferred route of administration, cost and access, insurance coverage, adverse-effect profile, comorbidities, and patient preferences (Figs. 4A and 4B) [4, 5, 44]. Shared decision-making should also explicitly address the probability that a given regimen will achieve the treatment goal. Expected treatment potency should be matched to the lipid-lowering gap, while the sequence of agents should be tailored to the treatment goal, specific risk category and statin-status pathway rather than a standardized universal approach. BA monotherapy typically lowers LDL-C by approximately 20–23%, and ezetimibe produces a similarly modest reduction. The combination of BA and ezetimibe generally lowers LDL-C by approximately 35%–40%, whereas PCSK9 directed therapies typically produce reductions of approximately 50%–60%. Intensive multidrug regimens combining therapies may reduce LDL-C by as much as 80%–85%, depending on the baseline regimen and the specific agents used (Table 3) [4, 5, 45–52]. Because treatment response varies according to background therapy and individual factors, lipoprotein levels should be reassessed periodically after treatment initiation, and therapy should be adjusted as needed.

**Table 3:** Effects of combination lipid-lowering therapies including bempedoic acid on Low density Lipoprotein-Cholesterol (LDL-C), non-high density lipoprotein (Non-HDL), and high-sensitivity C-reactive protein (hs-CRP)

Lipid Lowering Therapy	LDL-C ↓	Non-HDL ↓	hs-CRP ↓
Bempedoic acid^*^	22.94%^42^	18.17%^42^	−27.03%^42,47^
Ezetimibe + Bempedoic acid^*^	37.82%^43^	18.36%^43^	−30.48%^43,47^
Statin + Bempedoic Acid^**^	20.02%^43^	13.57%^43^	−20.72%^43,47^
Statin + Ezetimibe + Bempedoic acid^***^	63.6%^44^	60%^44^	47.7%^44,47^
PCSK9mAB + Bempedoic acid****	~ 66.5%^44–46^	~ 59.1%^44–46^	~ 28%^44–47^
PCSK9mAB + Ezetimibe + Bempedoic acid	~ 75%^@^	~ 55–65%^&^	~ 27%^&^
Statin + Ezetimibe + Bempedoic acid + PCSK9mAB	~ 86%^@^	~ 75–80%^#^	~ 40–50%^#^

^*^Compared with placebo

^**^Pooled effect across studies including a range of statin intensities and comparator groups

^***^Randomized controlled trial evaluating bempedoic acid 180 mg, ezetimibe 10 mg, and atorvastatin 20 mg

^****^Previous randomized trials showed that bempedoic acid provided an additional 30.3% reduction in LDL-C, 24.2% reduction in non-HDL cholesterol, and 28.5% reduction in hs-CRP compared with placebo in patients already receiving PCSK9 inhibitor therapy.^45^ Based on published evidence, PCSK9 inhibitors alone are expected to reduce LDL-C by approximately 52% and non-HDL cholesterol by approximately 46%, with little or no clinically meaningful effect on hs-CRP.^46,47^ Assuming these lipid-lowering effects are sequential (multiplicative rather than additive), the expected combined reductions are approximately 66.5% for LDL-C, 59.1% for non-HDL cholesterol, and 28.5% for hs-CRP

@ Combined therapies impact on LDL-C (~ 66%) was derived from the 2025 Focused Update of the 2019 ESC/EAS Guidelines for the management of dyslipidaemias (Fig. 2)^5^

^&^ Approximate combined non-HDL-C reduction (~ 60%) was estimated by applying the published effects of PCSK9 inhibitors (~ 50%) and Nexlizet® (bempedoic acid + ezetimibe; ~ 33%) sequentially (multiplicative model).^46,48^ Because PCSK9 inhibitors and ezetimibe have negligible effects on hs-CRP, the estimated hs-CRP reduction was assumed to be driven by bempedoic acid^47^

^#^ Approximate combined non-HDL-C reduction (~ 75–80%) was estimated by integrating the published non-HDL-C–lowering effects of high-intensity statins (~ 50%),^4^ Nexlizet® (~ 33%), and PCSK9 inhibitors (~ 50%) using a sequential (multiplicative) model.^46,48^ The estimated hs-CRP reduction (%) reflects the additive anti-inflammatory effects of high-intensity statins and bempedoic acid, whereas PCSK9 inhibitors and ezetimibe were assumed to have minimal impact on hs-CRP^47^

Proprotein convertase subtilisin/kexin type 9 monoclonal antibodies (PCSK9mAB)

Primary-prevention therapy should follow both the risk category and established statin status (Fig. 4A). For borderline/intermediate risk as determined by PREVENT-ASCVD (3 to < 10%), moderate-intensity statin therapy is reasonable at borderline risk and recommended at intermediate risk when tolerated. With confirmed intolerance, a reduced statin dose should be preserved if possible; adding ezetimibe and/or BA is reasonable, with no guideline-mandated sequence between them. High-risk primary prevention (PREVENT-ASCVD ≥ 10%) prioritizes high-intensity statin therapy when tolerated, followed by the addition of ezetimibe; if goals remain unmet, BA or PCSK9-directed therapy should be considered. Among patients with statin intolerance, preserving any tolerated statin is also beneficial, while BA and/or ezetimibe or PCSK9-directed therapy may be added when warranted by the gap to the target goal. CLEAR Outcomes serves as an evidence anchor because it enrolled selected high-risk, statin-intolerant primary prevention patients. These findings may be considered in similar circumstances; however, limited data are available on the reproducibility of benefit in lower risk patients because the trial did not evaluate a general borderline/intermediate risk primary-prevention population [4, 5, 31, 44]. Nevertheless, when an oral combination is considered, the fixed-dose combination of BA 180 mg and ezetimibe 10 mg may reduce pill burden. In a previously reported high-risk population receiving maximally tolerated statin therapy, this combination produced an additional placebo-corrected LDL-C reduction of approximately 38% at 12 weeks [53]. Whether to start PCSK9 directed therapy will depend on the residual gap to be addressed and the statin status seen in Fig. 4A.

Secondary-prevention therapy should follow both determining if at very-high-risk ASCVD and statin status (Fig. 4B). Patients with prior clinical ASCVD not at very high risk should be attempted to use maximally tolerated statin (if partial intolerance).If the patient remains above goal, adding ezetimibe, a PCSK9 monoclonal antibody, or BA according to the remaining LDL-C gap and patient preference is indicated. In statin intolerants, also attempting to retain a reduced statin dose if possible is beneficial, as well as initiating early combination with BA and/or ezetimibe, or PCSK9-directed therapy, alone or in combination, according to the gap. In very-high-risk ASCVD patients, high-intensity/maximally tolerated statin therapy also remains the backbone; if the patient remains above goal, prioritizing adding early ezetimibe and/or BA in combination with PCSK9-directed therapy is reasonable. In the corresponding intolerance pathway, preserve any tolerated statin therapy and use BA, ezetimibe, or PCSK9-directed therapy, alone or in combination. As mentioned, combination therapy should be initiated early when monotherapy is unlikely to reach the goal; a sequential approach remains reasonable when preferred by the patient, but providing close reassessment and timely escalation is recommended. Such intensification concern remains underlooked, as demonstrated in the GOULD registry [54]. Where, among patients with established ASCVD, only 17.1% underwent lipid-lowering treatment intensification over 2 years, while nearly two-thirds continued to have LDL-C levels above 70 mg/dL [54]. Treatment gaps may be particularly more consequential among statin-intolerant patients managed by clinicians who are less familiar with alternative lipid-lowering therapies.

Within Figs. 4A and 4B, PCSK9-directed therapies include evolocumab and alirocumab; inclisiran is positioned separately in selected secondary-prevention pathways. Current U.S. guidelines consider inclisiran when PCSK9 monoclonal antibodies cannot be tolerated or obtained, or when less-frequent dosing is strongly preferred [4]. In the secondary-prevention pathway for patients with statin intolerance, current guidance positions inclisiran after risk-based goals remain unmet on BA, with or without ezetimibe. Because its current position reflects the evidence available when the guideline was written, this rationale should be reconsidered when results of the ORION-4 trial (NCT03705234) [55] becomes available. This trial is evaluating the effect of inclisiran on cardiovascular outcomes. Similarly, enlicitide (the first oral PCSK9-inhibitor) has been thoroughly assessed in the CORALreef program (HeFH [heterozygous familial hypercholesterolemia], ASCVD [secondary prevention] and AddOn [high-risk primary or secondary prevention on statins]) [56–58] and is currently being evaluated for cardiovascular outcomes in the CORALreef Outcomes trial (NCT06008756) [59].

Patients who decline statin therapy without demonstrated intolerance should return to shared decision-making; if they continue to decline another trial, nonstatin therapy should be considered and tailored on an individualized basis. However, refusal should not be relabeled as intolerance or treated as a BA-specific guideline indication. The lipid profile within 4–12 weeks after treatment initiation or dose adjustment and every 6–12 months once goals are reached, with further intensification if risk-based goals remain unmet (Figs. 4A and 4B).

## Real-World Implementation Considerations

Routine implementation of BA is influenced by several practical considerations, including cost, access, and insurance coverage. In the United States, the average retail cost is approximately $500 per month; however, discount programs such as GoodRx® may reduce the monthly cost to approximately $230 [60]. Manufacturer-reported data indicate that Medicare beneficiaries pay an average of approximately $37 per month, with coverage approval reported in up to 87% of cases [61]. For eligible commercially insured patients, co-pay assistance programs may further reduce out-of-pocket costs to as little as $10 per month. Despite expanding insurance coverage, prior authorization requirements remain burdensome and may delay treatment initiation [62].

From an economic perspective, at its listed retail price, BA exceeds conventional cost-effectiveness thresholds with an incremental cost-effectiveness ratio (ICER) of approximately $166,800 per quality-adjusted life-year (QALY). However, with real-world pricing and available discount programs, ICER estimates decrease to approximately $47,000–$100,000 per QALY, falling within the commonly accepted willingness-to-pay threshold of $150,000 per QALY. Cost-effectiveness is further improved when BA is combined with ezetimibe, with reported ICERs of approximately $40,000 per QALY [63].

Compared with other lipid-lowering therapies, BA currently occupies an intermediate-to-high price range. It remains substantially more expensive than generic statins (e.g., atorvastatin, approximately $10–$20 per month) [64] and ezetimibe (approximately $25–$35 per month) [65].However, discounted BA prices available through GoodRx® overlap with the lower end of the price range for PCSK9i, with discounted evolocumab prices as low as approximately $239 per month [66].Nevertheless, PCSK9i have broader insurance coverage, with manufacturer-reported access for approximately 98% of commercially insured patients, 95% of Medicaid beneficiaries, and 84% of Medicare beneficiaries, although prior authorization is frequently required [67]. Consequently, the relative affordability of these therapies depends largely on insurance coverage, prior authorization approval, and the availability of financial assistance programs.

As an oral, once-daily therapy, BA provides a convenient alternative for patients who prefer to avoid injectable medications. However, real-world evidence suggests that injectable PCSK9i may achieve greater treatment persistence and adherence than oral lipid-lowering therapies, such as statins and ezetimibe, potentially owing to less frequent dosing and more structured follow-up [68, 69]. Despite these advantages, injectable therapies remain subject to patient- and system-level barriers, including injection discomfort, needle aversion, storage requirements, and appropriate administration training, all of which may hinder their widespread adoption [70]. The availability of oral PCSK9i therapies may shift the balance and remove the route of administration as a barrier in treatment discussions. However, the considerations discussed in this review and current guidelines would still apply when choosing therapy.

## Conclusions

BA is an effective oral lipid-lowering option for patients who do not achieve risk-based lipid goals with tolerated statin therapy or who are unable or unwilling to take a statin. Cardiovascular outcomes data from the CLEAR Outcomes trial support the use of BA in statin-intolerant patients with established ASCVD or at high cardiovascular risk who have LDL-C ≥ 100 mg/dL. However, its cardiovascular benefit in low- to intermediate-risk primary-prevention populations has not been directly established. In appropriately selected patients, BA may nevertheless be considered based on its anticipated effects on LDL-C, non-HDL-C and hs-CRP, within a guideline-directed framework and through shared decision-making. In patients with partial statin intolerance, the initial priority should be to preserve any tolerated statin exposure. Treatment should then be tailored using BA as monotherapy or in combination with other oral or injectable agents according to the residual therapeutic gap, risk-based treatment thresholds, preferred route of administration, medication access and affordability, and the patient’s adverse-effect profile. Clinicians should counsel and monitor patients for potential adverse effects, including hyperuricemia, gout, and tendon injury, particularly when relevant risk factors are present. This approach still positions BA still as a valuable component of individualized combination lipid-lowering therapy without extending its use beyond the available evidence.

## Key References

- Blumenthal RS, Morris PB, Gaudino M, Johnson HM, Anderson TS, Bittner VA, et al. 2026 ACC/AHA/AACVPR/ABC/ACPM/ADA/AGS/APhA/ASPC/NLA/PCNA Guideline on the Management of Dyslipidemia. JACC. 2026 May;87(19):2624–757. https://doi.org/10.1016/j.jacc.2025.11.016
  - ○ New guidelines that redefined clinical practice and thresholds for the management of dyslipidemias, clinical algorithms are based on these guidelines.
- Nissen SE, Lincoff AM, Brennan D, Ray KK, Mason D, Kastelein JJP, et al. Bempedoic Acid and Cardiovascular Outcomes in Statin-Intolerant Patients. N Engl J Med. 2023 Apr 13;388(15):1353–64. https://doi.org/10.1056/NEJMoa2215024
  - ○ Landmark outcomes trial, that established bempedoic acid as the only oral therapy that demonstrated positive cardiovascular outcomes in patients living with statin intolerance.
- Cheeley MK, Saseen JJ, Agarwala A, Ravilla S, Ciffone N, Jacobson TA, et al. NLA scientific statement on statin intolerance: a new definition and key considerations for ASCVD risk reduction in the statin intolerant patient. J Clin Lipidol. 2022 Jul;16(4):361–75. https://doi.org/10.1016/j.jacl.2022.05.068
  - ○ Comprehensive definition of statin intolerance by the national lipid association. The definition of statin intolerance in this manuscript is based on this reference.

## Data Availability

No datasets were generated or analysed during the current study.

## Abbreviations

**ACL**: ATP-citrate lyase
**ACSVL1**: Very long-chain acyl-CoA synthetase 1
**apoB**: Apolipoprotein B
**ARR**: Absolute risk reduction
**ASCVD**: Atherosclerotic cardiovascular disease
**ATP**: Adenosine triphosphate
**AU**: Agatston units
**BA**: Bempedoic acid
**BMI**: Body mass index
**BUN**: Blood urea nitrogen
**CAC**: Coronary artery calcium
**CI**: Confidence interval
**CK**: Creatine kinase
**CKD**: Chronic kidney disease
**CLEAR**: Cholesterol Lowering via Bempedoic Acid, an ACL-Inhibiting Regimen
**CVD**: Cardiovascular disease
**eGFR**: Estimated glomerular filtration rate
**FDA**: U.S. Food and Drug Administration
**GOULD**: Getting to an Improved Understanding of Low-Density Lipoprotein Cholesterol and Dyslipidemia Management
**HbA1c**: Hemoglobin A1c
**HeFH**: Heterozygous familial hypercholesterolemia
**HMG-CoA**: 3-Hydroxy-3-methylglutaryl coenzyme A
**HR**: Hazard ratio
**hs-CRP**: High-sensitivity C-reactive protein
**ICER**: Incremental cost-effectiveness ratio
**LDL-C**: Low-density lipoprotein cholesterol
**MACE**: Major adverse cardiovascular events
**MACE − 3**: Three-component major adverse cardiovascular event composite
**MACE − 4**: Four-component major adverse cardiovascular event composite
**MI**: Myocardial infarction
**NCATS**: National Center for Advancing Translational Sciences
**NIH**: National Institutes of Health
**NLA**: National Lipid Association
**NNT**: Number needed to treat
**non-HDL-C**: Non-high-density lipoprotein cholesterol
**OAT2**: Organic anion transporter 2
**Non-HDL-C**: Non-high-density lipoprotein cholesterol
**PCSK9**: Proprotein convertase subtilisin/kexin type 9
**PCSK9i**: Proprotein convertase subtilisin/kexin type 9 inhibitor
**PREVENT-ASCVD**: Predicting Risk of Cardiovascular Disease Events Atherosclerotic Cardiovascular Disease Risk Estimator
**QALY**: Quality-adjusted life-year
**RRR**: Relative risk reduction
**SAMSON**: Self-Assessment Method for Statin Side-effects Or Nocebo
**SAMS**: Statin-associated muscle symptoms
**SCORE**: Systematic Coronary Risk Evaluation
**siRNA**: Small interfering RNA
**ULN**: Upper limit of normal
